# Reliability and validity of a Global Physical Activity Questionnaire adapted for use among pregnant women in Nepal

**DOI:** 10.1186/s13690-023-01032-3

**Published:** 2023-02-10

**Authors:** Noha Algallai, Kelly Martin, Krupali Shah, Kusum Shrestha, Jean-Francois Daneault, Archana Shrestha, Abha Shrestha, Shristi Rawal

**Affiliations:** 1 Department of Clinical and Preventive Nutrition Sciences, School of Health Professions, Rutgers the State University of New Jersey, Newark, NJ USA; 2 Department of Human Ecology, SUNY Oneonta, Oneonta, NY USA; 3 Department of Public Health, Kathmandu University School of Medical Sciences, Dhulikhel, Nepal; 4 Department of Rehabilitation and Movement Sciences, School of Health Professions, Rutgers the State University of New Jersey, Newark, NJ USA; 5 Department of Chronic Disease and Epidemiology, Center of Methods for Implementation and Prevention Science, Yale School of Public Health, New Haven, CT USA; 6 Institute for Implementation Science and Health, Kathmandu, Nepal; 7 Department of Obstetrics and Gynecology, Dhulikhel Hospital, Dhulikhel, Nepal

**Keywords:** Physical activity, Maternal health, Accelerometer, GPAQ, Validation, Pregnancy

## Abstract

**Background:**

Physical activity (PA) plays an important role in optimizing health outcomes throughout pregnancy. In many low-income countries, including Nepal, data on the associations between PA and pregnancy outcomes are scarce, likely due to the lack of validated questionnaires for assessing PA in this population. Here we aimed to evaluate the reliability and validity of an adapted version of Global Physical Activity Questionnaire (GPAQ) among a sample of pregnant women in Nepal.

**Methods:**

A cohort of pregnant women (*N=*101; age 25.9±4.1 years) was recruited from a tertiary, peri-urban hospital in Nepal. An adapted Nepali version of GPAQ was administered to gather information about sedentary behavior (SB) as well as moderate and vigorous PA across work/domestic tasks, travel (walking/bicycling), and recreational activities, and was administered twice and a month apart in both the 2^nd^ and 3^rd^ trimesters. Responses on GPAQ were used to determine SB (min/day) and total moderate to vigorous PA (MVPA; min/week) across all domains. GPAQ was validated against PA data collected by a triaxial accelerometer (Axivity AX3; UK) worn by a subset of the subjects (*n=*21) for seven consecutive days in the 2^nd^ trimester. Intra-class correlation coefficients (ICC) and Spearman’s rho were used to assess the reliability and validity of GPAQ.

**Results:**

Almost all of the PA in the sample was attributed to moderate activity during work/domestic tasks or travel. On average, total MVPA was higher by 50 minutes/week in the 2^nd^ trimester as compared to the 3^rd^ trimester. Based on the World Health Organization (WHO) guidelines, almost all of the participants were classified as having a low or moderate level of PA. PA scores for all domains showed moderate to good reliability across both the 2^nd^ and 3^rd^ trimesters, with ICCs ranging from 0.45 (95%CI: (0.17, 0.64)) for travel PA at 2^nd^ trimester to 0.69 (95%CI: (0.51, 0.80)) for travel PA at 3^rd^ trimester. Reliability for total MVPA was higher in the 3^rd^ trimester compared to 2^nd^ trimester [ICCs 0.62 (0.40, 0.75) vs. 0.55 (0.32, 0.70)], whereas the opposite was true for SB [ICCs 0.48 (0.19, 0.67) vs. 0.64 (0.46, 0.76)]. There was moderate agreement between the GPAQ and accelerometer for total MVPA (rho = 0.42; *p* value <0.05) while the agreement between the two was poor for SB (rho= 0.28; *p* value >0.05).

**Conclusions:**

The modified GPAQ appears to be a reliable and valid tool for assessing moderate PA, but not SB, among pregnant women in Nepal.

## Background

Physical activity (PA) plays an important role in optimizing health outcomes throughout pregnancy. The World Health Organization (WHO) suggests that pregnant women participate in at least 150 minutes of moderate intensity PA per week [1], as this may be protective against adverse pregnancy outcomes including excessive gestational weight gain and gestational diabetes mellitus [2, 3]. PA that incorporates both aerobic and resistance exercises can positively impact cardiorespiratory health during pregnancy [4], and PA is generally related to greater overall health and quality of life among adults [5, 6]. Valid and reliable tools are essential to assess PA patterns and their relationship to health outcomes in pregnancy [7]. In population-based studies, questionnaires are the most commonly used instrument to assess and measure PA, as they are relatively quick, low-cost, and easy to administer among large populations [7]. In many low-income countries, including Nepal, data on the associations between PA and pregnancy outcomes are scarce, likely due to the lack of validated questionnaires for assessing PA in this population.

Globally, multiple questionnaires have been utilized to assess PA during pregnancy, including the Pregnancy Physical Activity Questionnaire (PPAQ) [8], the International Physical Activity Questionnaire (IPAQ) [9], and the Global Physical Activity Questionnaire (GPAQ) [10]. The PPAQ was developed and evaluated for use during pregnancy [8], and has shown adequate validity and reliability across pregnant populations in several countries [11], though its applicability within low to middle-income countries (LMICs) remains unknown. The IPAQ was developed in the late 1990s to help standardize the measurement of health-related PA across 12 countries [9], but was found to have poor agreement and correlation with accelerometry measures during pregnancy [9]. In 2002, the WHO created the GPAQ, a modified version of the IPAQ, in response to a greater cross-cultural interest in understanding the relationship between PA and health outcomes [10]. The GPAQ has the advantage of capturing and assessing self–reported PA across three domains: work/domestic tasks, travel/transportation, and leisure/recreation tasks. The WHO specifically developed the GPAQ for PA surveillance in LMICs [10], where it has since been extensively used and validated [12, 13], including among non-pregnant adults within Nepal [9, 10, 14–16]. While the GPAQ has been used to assess PA during pregnancy in a few studies, [17, 18] validation studies in pregnant populations remain limited [19].

Notably, many studies assessing PA during pregnancy have not utilized validated measurement tools when evaluating associations between PA and health outcomes [20]. It is important that self-report PA questionnaires, such as the GPAQ, are validated against objective measures of PA, such as accelerometry, within each specific population that they will be utilized [7]. Currently, the validity of the GPAQ has only been explored among a single population of pregnant women in South Africa, where it was found to overestimate PA and underestimate sedentary behavior (SB) [19]. Here, we aimed to evaluate the reliability and validity of an adapted version of the GPAQ among pregnant women in a periurban setting in Nepal.

## Methods

### Study design

This prospective cohort study recruited 101 pregnant women attending the Obstetric Outpatient Department (OPD) at Dhulikhel Hospital in Dhulikhel**, **Nepal, for antenatal care (ANC) between January 2019 and January 2020. Women were enrolled during their 1^st^ trimester ANC visit (5-14 weeks of gestation) and followed through the 2^nd^ and 3^rd^ trimester, up until six weeks postpartum. The primary aim of the study was to adapt and validate dietary and PA assessment tools for use among Nepalese pregnant women. With respect to PA, the study aimed to evaluate the reliability and validity of a culturally adapted version of the 16-item GPAQ [10]. Because PA may vary dramatically during pregnancy due to physiological changes, we assessed its reliability at two-time points in the 2^nd^ and 3^rd^ trimester of pregnancy. Accelerometer data from the 2^nd^ trimester was used for validation as pregnant women are more likely to be active during this time [9].

To participate, women had to be 18 years or older, ≤14 weeks of gestation at enrollment, and carrying a single fetus. Additionally, to participate in the validation study with the accelerometer in the 2^nd^ trimester, the participant had to agree to wear the accelerometer on their wrist for seven consecutive days; women with contraindications to exercise were not eligible for the validation study. At enrollment and subsequent ANC visits, a trained research assistant collected data from each participant via medical record review and patient interview. Data was collected on socio-demographic, lifestyle, and clinical characteristics. All participants provided written informed consent. The Kathmandu University Ethical Review Board (102/18) and the Rutgers Newark Health Sciences Institutional Review Board (Pro2018001976) approved the study protocol.

### GPAQ adaptation for the target population

The GPAQ includes 16 questions which gather information about SB as well as levels of PA across five settings. These include PA related to work/domestic/occupational tasks (vigorous and moderate), travel/transportation such as walking or biking, and leisure/recreational activities (vigorous and moderate) [9, 10, 14–16]. Vigorous activity is defined as an activity that causes large increases in breathing or heart rate while moderate activity causes small increases in heart rate.

For this study, a modified GPAQ was developed by translating the questionnaire into Nepali and creating a PA chart that included examples specific to the target demographic of Nepali pregnant women. For PA related to work/domestic tasks, examples for vigorous activity included construction work and carrying/stacking heavy loads (i.e., bricks), whereas examples for moderate activity included farm work, drawing water from well, and carrying light loads. For leisure/recreational PA, examples for vigorous activity included running and hiking/trekking, while examples for moderate activity included swimming or yoga. For each of the five PA domains (work-related vigorous, work-related moderate, travel, recreational vigorous, and recreational moderate), the GPAQ asks participants to report the time duration and frequency (number of days per week) of these activities during a typical week. To ascertain SB, participants are asked to report the amount of time they spent sitting per typical day (i.e., watching television/lounging on the couch/sitting at work). To adapt the questionnaire for use in pregnancy, we further specified the timeframe so that it refers to the PA during a typical week in the respective trimester (2^nd^ or 3^rd^). The questionnaire was back translated in order to ensure fidelity between the English and Nepali versions. The Nepali version of the questionnaire was also cognitively tested among 8 pregnant volunteers to ensure its suitability in capturing the usual types and duration of physical activity among our target population.

### GPAQ administration

To determine the trimester-specific reliability of the GPAQ, the questionnaire was administered to participants at two time-points, one month apart, in the 2^nd^ and 3^rd^ trimesters. Despite being administered a month apart, at both time points, the questions on the GPAQ were directed to ascertain PA and SB during a typical week in the respective trimester (2^nd^ or 3^rd^ trimester). Responses on the GPAQ were used to determine the duration of SB (min/day), as well as PA (minutes/week) for each of the five domains (work-related vigorous, work-related moderate, travel, recreational vigorous, and recreational moderate). Total moderate or vigorous PA (MVPA in min/week) was calculated by adding time spent in minutes/week across all five domains.

**Fig. 1:**
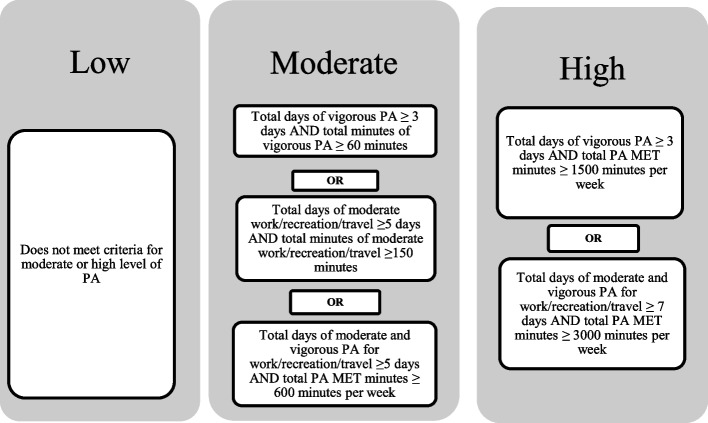
Classification of PA Level based on WHO Guidelines. Shows the specific criteria for low, moderate, and high physical activity classifications based on the World Health Organization standards. Abbreviations: PA; physical activity, MET; metabolic equivalent

The GPAQ also allows for the computation of energy expenditure, recorded in metabolic equivalent tasks (METs). A standard value of 4 METs was assigned for moderate-intensity activities related to work/recreation/travel; 8 METs was assigned for work-or leisure-related vigorous activity [21]. According to the GPAQ responses with respect to duration, intensity, and frequency of activity, total MVPA was also calculated in MET-min/week. Total MVPA level was also classified as low, moderate, and high based on WHO guidelines (Fig. 1) [21].

### Accelerometer

Data collected from the GPAQ was validated against PA data collected by a triaxial accelerometer (Axivity AX3; UK). The device captured activity based on changes in acceleration using a three-dimensional (3D) acceleration sensor, providing a more accurate and objective calculation of energy expenditure as compared to uniaxial devices and self-reported PA [22]. A subset of the subjects (*n=*34) who agreed to take part in the validation study wore the Axivity accelerometer on their left wrist for seven consecutive days during their 2^nd^ trimester.

The accelerometer was set to record 3D acceleration data at a sample rate of 50Hz with a dynamic range of ±8g. The activity classification and non-wear time for each participant was extracted using the UK Biobank accelerometer analysis tool [23]. Non-wear time was defined as at least 60 consecutive minutes of stationary episodes with a standard deviation of less than 13.0mg and was imputed with data from similar time-of-day vector magnitude. For this analysis, MVPA was defined using a 100mg cut-off, as is done traditionally; and average daily time in MVPA (min/day) and SB (min/day) were calculated. All accelerometer data analysis was performed in Python. A minimum of 72 hours of continuous and valid wear time was necessary for inclusion in the analysis; 21 of 34 participants were included in the final analyses [23].

### Statistical analysis

Descriptive statistics are presented as mean ±SD or median (25^th^-75^th^ percentiles) for continuous variables, and frequencies (n, %) for categorical variables. The reliability of the GPAQ between the two visits within each trimester was tested by intra-class correlation coefficients (ICC) and Spearman's correlation coefficients (rho) for continuous variables including PA measures in each of the five domains (min/week), total MVPA (min/week), and SB (min/day). With respect to the level of PA (i.e. high, moderate, or low), the degree of consistency between the two GPAQ administrations in each trimester was evaluated using the Gwet's agreement coefficient (AC_1_). Gwet's AC_1_ was used because a skewed distribution was observed and the AC_1_ statistic overcomes the noted limitations of the kappa statistic and provides a more precise, stable agreement statistic [24]. For validation analyses comparing the average GPAQ measures in the 2^nd^ trimester with those from the accelerometer, we used Spearman's correlation coefficients (rho) for continuous variables including total MVPA and SB.

ICC and Gwet's AC1 analyses were interpreted based the Cicchetti and Sparrow (1981) criteria [25]: <0.40 poor, 0.41-0.59 fair/moderate, 0.60-0.74 good and >0.75 excellent reliability. To interpret Spearman’s rho, the following criteria were used: 0-0.20 poor, 0.21-0.40 fair, 0.41-0.60 moderate/acceptable, 0.61-0.80 good, and 0.81-1.00 strong correlation [26]. 

## Results

Demographic characteristics of the sample are outlined in Table 1. Out of 101 participants enrolled at baseline, 90 and 83 participants completed both visits in the 2^nd^ and 3^rd^ trimester respectively. On average, the two visits in the 2^nd^ trimester as well as the 3^rd^ trimester were 4 weeks apart, and the mean difference in gestational age between 2^nd^ trimester 1st visit and 3^rd^ trimester 2^nd^ visit was about 16 weeks. There were no significant differences in demographic and clinical characteristics between women who did and did not complete the follow-up visits in the 2^nd^ and 3^rd^ trimesters. The primary reason for attrition was loss of contact (e.g., phone switched off, change in telephone number); only 2 had confirmed pregnancy loss.

**Table 1:** Demographic, lifestyle, and clinical characteristics of study participants (*N=*101)

**Variables**	**Mean± SD**
**Age (Years)**	25.9 ± 4.1
**Pre-pregnancy BMI (Kg/m**^**2**^**)**	24.3 ± 3.7
**Gestational Age**	
At 2^nd^ trimester Visit 1 (weeks)	17.7 ± 2.2
At 2^nd^ trimester Visit 2 (weeks)	22.2 ± 2.2
At 3^rd^ trimester Visit 1 (weeks)	29.9 ± 1.7
At 3^rd^ trimester Visit 2 (weeks)	34.5 ± 1.9
**Pre-pregnancy BMI**	**N (%)**
Underweight (BMI <18.5kg/m^2^)	4 (4.0)
Normal Weight (BMI 18.5-24.9kg/m^2^)	56 (55.4)
Overweight (BMI 25.0-29.9kg/m^2^)	34 (33.7)
Obese (BMI ≥30.0kg/m^2^)	7 (6.9)
**Educational Level**	
Less than 10 years	16 (15.1)
10 years or more	85 (80.2)
**Ethnic Group**	
Brahmin	22 (21.8)
Chettri/Thakuri/Sanyasi	16 (15.8)
Newar	39 (38.6)
Magar/Tamang/Rai/Limbu	20 (19.8)
Kami/Dami/Sarki/Gaaine/Baadi	4 (4.0)
**Employment Status**	
Homemaker	56 (55.4)
Non-government employee	18 (17.8)
Self-employed	15 (14.9)
Student	7 (6.9)
Government employee3 (3.0)	3 (3.0)
Non-paid	2 (2.0)
**Income level (Annual, Nepali Rupees)**	
10,000 – 30,000	3 (3.0)
30,000 – 50,000	58 (57.4)
>50,000	40 (39.6)
**Smoking use before the pregnancy**	
Yes	2 (2.0)
No	99 (98.0)
**Alcohol use before pregnancy**	
Yes	29 (27.9)
**Alcohol intake before pregnancy (mL/day)**	4.3 ±9.1

Key: *BMI* Body mass index, *kg* Kilogram, *m* Meters, *N* Number, *SD* Standard deviation; %, percent

Levels of PA as assessed by the GPAQ can be found in Table 2. None of the participants reported vigorous PA, either work-related or recreational, and only one participant reported moderate recreational PA. Almost all of the PA in the sample was attributed to moderate activity at home, work, or in travel-related tasks. Both work-related moderate PA and travel-related PA were higher in the 2^nd^ trimester than the 3^rd^ trimester; on average, total MVPA was higher by 50 min/week in the 2^nd^ trimester. Mean reported time for SB declined by 27 min/week from the 2^nd^ trimester to the 3^rd^ trimester, yet the median values were consistent across four visits. Based on the WHO guidelines, almost all participants were classified as having low or moderate levels of PA, with the latter accounting for majority of the participants. The proportion of participants with moderate level of PA declined from the 2^nd^ trimester to the 3^rd^ trimester.

**Table 2:** Reliability estimates for the GPAQ: Intraclass and Spearman's correlation across two visits in the 2nd trimester (*n=* 92) and 3rd trimester (*n=* 84) of pregnancy

**PA Measure**	**2**^**nd**^** trimester**				**3**^**rd**^** trimester**			
	**1**^**st**^** visit** **(Mean ± SD)**	**2**^**nd**^** visit** **(Mean ± SD)**	**ICC (95%CI)**	**Spearman’s Rho (95%CI)**	**1**^**st**^** visit** **(Mean ± SD)**	**2**^**nd**^** visit** **(Mean ± SD)**	**ICC (95%CI)**	**Spearman’s Rho (95%CI)**
Work-related Moderate PA (min/week)	167.8± 115.9	135.9 ±98.9	0.67 (0.50, 0.78)**	0.59 (0.38, 0.74)**	113.9 ±95.9	123.4 ±125.1	0.62 (0.41, 0.76)**	0.62 (0.44, 0.76)**
^a^Travel-related PA (min/week)	132.6± 108.4	134.8 ±110.1	0.45 (0.17, 0.64)*	0.35 (0.14, 0.54)*	125.8 ±98.2	114.9 ± 97.5	0.69 (0.51, 0.80)**	0.49 (0.28, 0.67)**
Sedentary Behavior (min/day)	133.7± 71.8	119.6 ±59.8	0.64 (0.46, 0.76)**	0.50 (0.31, 0.66)**	103.5 ± 48.2	99.4 ± 37.5	0.48 (0.19, 0.67)*	0.36 (0.13, 0.55)*
Total MVPA (min/week)	300.3± 144.2	270.8±147.6	0.55 (0.32, 0.70)**	0.45 (0.25, 0.61)**	241.6 ±131.5	238.3 ± 146.2	0.62 (0.40, 0.75)**	0.46 (0.25, 0.62)**
Total MVPA (MET-min/week)	1201.4± 576.7	1083.2± 590.5			966.2 ± 525.9	953.3 ± 584.9		
**PA level**	**n (%)**	**n (%)**	**Gwet's AC1** **(95% CI)**	**% Agreement**	**n (%)**	**n (%)**	**Gwet's AC1 (95% CI)**	**% Agreement**
^b^Low	27 (29.3%)	29 (32.2%)	0.61 (0.49, 0.74)	70.0%	29 (34.5%)	33 (39.8%)	0.60 (0.47, 0.74)	69.6%
^c^Moderate	64 (69.6%)	60 (66.7%)			55 (65.5%)	49 (59 %)		
^d^High	1 (1.1%)	1 (1.1 %)			-	1 (1.2%)		

**Note:** There were no participants in this study that reported vigorous activity either for work or recreational activity of the GPAQ. Only one participant reported moderate activity for leisure/recreation, hence data is not shown.

*CI* Confidence Interval, *GPAQ* Global Physical Activity Questionnaire, *ICC* Intra class correlation; task, *PA* Physical activity, *MET* Metabolic equivalent, *SD* Standard deviation

^*^p value <0.01

^**^p value <0.0001

^a^ Travel component includes PA from walking or biking

^*b*^ Low IF: the value does not reach the criteria for either high or moderate levels of physical activity.

^*c*^* Moderate* IF: (total physical activity vigorous days >= 3 days AND total physical activity vigorous >= 60 minutes OR IF: Moderate physical activity for work and recreation days+ travel days >= 5 days AND total moderate physical activity for work and recreation+ total travel>= 150 minutes OR IF: total vigorous and moderate physical activity days for work and recreation + travel days>= 5 days AND Total physical activity MET minutes per week >= 600

^*d*^ High IF:( total physical activity vigorous days) >= 3 days AND Total physical activity MET minutes per week is >= 1500 OR IF: Total physical activity for vigorous, moderate and travel days >= 7 days AND total physical activity MET minutes per week is >= 3000^20^

### Reliability of the GPAQ

All PA scores across the 5 domains showed moderate to good reliability estimates in the 2^nd^ and 3^rd^ trimester (Table 2). In the 2^nd^ trimester, the PA scores across 5 domains showed fair to moderate correlations (rhos ranging from 0.35 to 0.59), and moderate to good reliability with ICCs ranging from 0.45 to 0.67. In the 3^rd^ trimester, the PA scores across 5 domains showed fair to good correlations (rhos ranging from 0.36 to 0.62), and moderate to good reliability (ICCs ranging from 0.48 to 0.69). Reliability for total MVPA was higher in the 3^rd^ trimester compared to the 2^nd^ trimester (ICCs 0.62 vs. 0.55), whereas the opposite was true for SB (ICCs 0.48 vs. 0.64). Consistency across the WHO classifications of low, moderate, and high level of PA, as measured using the AC_1_ statistic, was 0.614 (95% CI: 0.485, 0.744) in the 2^nd^ trimester and 0.603 (95% CI: 0.469, 0.738) in the 3^rd^ trimester, indicating good reliability. The percentage agreement across the 3 categories were 70% and 69.6% for the 2^nd^ and 3^rd^ trimester, respectively.

### Validity of the GPAQ

GPAQ was validated against PA data collected by a 3D accelerometer worn by a subset of the subjects (*n=*21) for seven consecutive days in the 2^nd^ trimester. Construct validity was assessed by comparing MVPA and SB, reported with the GPAQ and those derived from accelerometer counts, respectively (Table 3). The mean minutes of MVPA reported on GPAQ was similar to that measured by accelerometer (104.8 minutes on GPAQ vs. 112.5 minutes by accelerometer). The mean minutes of SB per day reported on GPAQ was 127.1 minutes, which was on average 330 minutes lower compared to that measured by the accelerometer. There was moderate correlation (rho = 0.42) between the GPAQ and accelerometer data for MVPA at 2^nd^ trimester. However, there was only fair correlation (rho= 0.28) between SB assessed by the accelerometer and the GPAQ measure.

**Table 3:** Criterion validity of the GPAQ: Intraclass and Spearman's correlation between the GPAQ and accelerometer data at 2^nd^ trimester (*n =* 21)

**PA measure**	**GPAQ 2**^**nd**^** trimester/average**	**Accelerometer 2**^**nd**^** trimester**	**Spearman’s Rho (95% CI)**
**MVPA in the 2**^**nd**^** trimester (min/day)**	104.8 ± 47.2	112.5 ±35.5	0.42 (0.003,0.77)*
**SB in the 2**^**nd**^** trimester (min/day).**	127.1 ± 58.2	465.3 ± 122.5	0.28 (-0.17, 0.63)

Key: *ICC* Intra class correlation, *CI* Confidence Interval, *MET* Metabolic equivalent task, *MVPA* Moderate to vigorous physical activity, *SB* Sedentary behavior

^*^p value <0.05

## Discussion

This study examined the reliability and validity of a modified GPAQ that was adapted and translated for use among a sample of pregnant Nepalese women. The modified GPAQ appears to be a reliable and valid tool for assessing MVPA, but not SB, among pregnant women in Nepal. To our knowledge, this is the first PA assessment tool to be validated for use among pregnant women in Nepal.

Levels of PA among our sample were similar to those previously reported among samples of Asian women [27, 28]. A sample of pregnant women from Singapore (*N=*1171) reported an average of 1039.5 MET min/week between 26-28 gestation weeks, [27] while we reported 1142.3 min/week in the 2^nd^ trimester and 959.8 min/week in the 3^rd^ trimester. A relatively similar proportion of women were insufficiently active or had low activity (<600 MET min/week) in our sample (37.4%) compared to the sample in Singapore (34.1%) [27]. In another study with pregnant Taiwanese women, levels of household/occupational activity accounted for the highest percentage of PA performed across trimesters, [28] which is similar to our sample where almost all PA was attributed to moderate PA related to work/domestic tasks and travel. In addition, we observed an expected decline in all types of PA from early to late pregnancy, consistent with findings across populations of pregnant women from Asian [27] and non-Asian countries [29]. However, while prior studies also observed increases in SB across pregnancy, we found that the median SB levels remained consistent across four visits in pregnancy [27–29].

Our reliability and validity estimates were similar or slightly better than those reported among pregnant women in prior literature [11]. Of note, the validity of the GPAQ has only been explored among a single population of pregnant women in South Africa, where it was found to have poor agreement with accelerometry when measuring either PA or SB [19]. Specifically, Watson et al (2017) [19] found that the GPAQ had poor validity for total PA as well as SB, overestimating MVPA and underestimating SB when compared to accelerometer data [19]. The cultural and linguistic diversity of the South African study sample could have affected the internal validity of the study, and contributed to these negative findings. In our relatively homogeneous sample, the GPAQ underestimated SB but mean MVPA levels were similar across GPAQ and accelerometer measures. Among other studies utilizing the IPAQ and PPAQ there have been mixed results, with some finding acceptable validity and reliability and others finding reasonable reliability but poor validity, particularly for SB, and vigorous PA [9, 11, 30]. Harrison et al (2011) [9] found poor agreement between the IPAQ and the accelerometer data, with no significant correlation between the total, light, and moderate METminutes calculated using the accelerometer and those reported by the participants on the IPAQ (Harrison et al. 2011) [9]. Similar to Watson et al (2017)’s finding with GPAQ [19], Harrison et al (2011) [9] found that self-reported moderate METminutes were overestimated by the IPAQ when compared with the accelerometer; in contrast, the total METminutes and the light METminutes were underestimated [9]. Of note, this Australian study [9] consisted exclusively of women with overweight BMI status, who have been observed to over-report PA in some studies [37], which might explain the observed poor validity estimates. When adapting the PPAQ among a sample of pregnant women from China, Xiang et al (2016) [30] noted good reliability for total PA and sedentary, light, and moderate activity, but a lower reliability for vigorous activity [30]. In addition, the validity estimates were poor for both moderate and vigorous PA but were reasonable for light activity (*r=*0.33) and total PA (*r=*0.35) [30]. However, an important limitation of this study [30] was the use of uniaxial rather than triaxial accelerometry, which might have affected the validity estimates due to its limited measurement ability for certain PA activities (e.g., stationary exercises).

There are a few limitations to our study. Recall bias could have led to errors in self-report of PA behaviors, and we cannot rule out the potential for over or under estimation [31], though our shorter timeframe between GPAQ administrations is a desirable method to collect more accurate PA estimates from participants [9]. In addition, only a small subset of participants adhered to wearing the accelerometer for seven days. Compliance is a common concern among studies utilizing wearable accelerometer and mobile sensor technology [32]. While smartphones and their associated health applications are increasingly accessible, tracking PA via these devices is problematic as they may underestimate total steps and distance traveled when not routinely carried by the participant [33, 34]. In addition, while it is well-known that PA monitors are most accurate if placed on the hip, most wearable accelerometer devices are worn on the wrist and tend to misclassify non-ambulatory arm movements such as those completed in resistance or strength training exercises [32]. Still, accelerometers are notably more accurate than participant self-report even though few are thoroughly validated for these purposes [32]. With few women engaging in vigorous activity in our study, as in prior literature, [30, 35] we could not validate the GPAQ among our sample with respect to this type of exercise. Cultural attitudes surrounding exercise during pregnancy may explain why low levels of vigorous PA were observed among our sample [35]. Recommendation against vigorous activity during pregnancy is prevalent in Asian populations, as it is viewed as potentially causing stress and harm to the mother and developing baby [35]. Lastly, since this study was done in a small sample of women recruited from a single hospital in Nepal, our findings cannot be generalized with assurance to pregnant populations elsewhere.

Despite these limitations, there are several strengths to our study. Our study adhered to standardized WHO protocols in administering the GPAQ and was the first to establish the reliability and validity of the GPAQ in the 2^nd^ and 3^rd^ trimesters, which enhances our understanding regarding usability of this tool within the pregnant population. Our selection of the GPAQ was intentional, as it is a PA assessment tool that has been extensively applied within LMICs like Nepal [9, 15, 16, 36]. We validated data from the GPAQ against data collected by a 3D accelerometer, which is preferred over self-reporting of PA and uniaxial accelerometry as it provides a more accurate calculation of energy expenditure [22]. The GPAQ was interviewer-administered by the same trained staff throughout the study, enhancing the reliability of the data collected [31]. In addition, we adapted and translated the GPAQ to include culture-specific PA examples to improve the suitability of the questionnaire for our target population of pregnant Nepalese women.

## Conclusion

In this study, the GPAQ showed acceptable reliability and validity when used to assess moderate PA among a sample of pregnant women in Nepal. However, we recommend the GPAQ be used with caution to assess SB among pregnant women.

## Data Availability

The datasets generated and/or analyzed during the current study are not publicly available due to privacy and confidentiality reasons but are available from the corresponding author on reasonable request.

## Abbreviations

CI: Confidence Interval
GPAQ: Global Physical Activity Questionnaire
ICC: Intra class correlation
rho: Spearman's correlation coefficients
PA: Physical activity
MET: Metabolic equivalent
MVPA: Moderate to vigorous physical activity
SB: Sedentary behavior
SD: Standard deviation
